# Recent advances in psychological therapies for eating disorders

**DOI:** 10.12688/f1000research.7618.1

**Published:** 2016-04-19

**Authors:** Glenn Waller

**Affiliations:** 1 Clinical Psychology Unit, Department of Psychology, Western Bank, University of Sheffield, Sheffield, UK

**Keywords:** eating disorders, anorexia nervosa, anorexia, bulimia nervosa, bulimia

## Abstract

Recent years have seen substantial consolidation and development of the evidence base for psychological therapies for eating disorders. This review summarises the key changes over that time period. Specific forms of cognitive behavioural therapy and family-based treatment have consolidated and extended their positions as treatments of choice despite the development of novel approaches. However, there is still a significant need for further development and testing to improve recovery rates, particularly in anorexia nervosa.

## Recent advances in psychological therapies for eating disorders

How far have we progressed in the treatment of eating disorders during the current decade? In a previous review ^1^, it was suggested that developments were necessary. There has been little advance in some areas that were identified as targets for further research, such as treatment matching and the role of pharmaceutical interventions ^2^. However, there have been substantial developments in psychotherapies and their outcomes since 2009. This review will summarise the evidence relating to these advances.

The psychotherapies considered here are designed to treat the eating disorder in and of themselves. However, there are also symptom-based and adjunctive approaches that are designed to address specific elements of the eating disorder (e.g., cognitive flexibility) without the expectation that they will bring about remission on their own. Recent evidence regarding some of those approaches will be considered separately below.

## Psychotherapy outcomes: some consolidation, some change

### Therapeutic context

The setting in which psychological therapies is carried out is an important issue and is at least partly determined by local health practices. For example, in some countries, individuals with bulimia nervosa are routinely treated in a combination of in- and out-patient settings, whereas in others it is very rare for them to be treated as in-patients at all. Thus, findings need to be understood in their context. A particular issue in interpreting treatment outcomes is the need to understand the degree of in-patient work that has been involved in the treatment of patients with anorexia nervosa. In-patient care for anorexia nervosa is not a predictor of better outcomes than treatment in less intensive settings and is substantially more expensive ^3, 4^, suggesting that its use should be confined to medical need (e.g., preliminary weight gain, medical stabilisation). Similarly, there is little to suggest that any specific psychotherapy is more effective in an in-patient setting.

Another issue is whether the delivery modality makes a difference in terms of outcomes. In short, there has been little change here. Different forms of self-help and group treatments are less effective than face-to-face individual therapy, as has been the case since the different modalities were developed. More recent work has examined the potential of electronic media (e.g., smartphone apps) for delivering therapy. However, to date, there is little robust evidence that this is an effective approach ^5, 6^. Therefore, unless otherwise specified, it should be noted that the following conclusions usually are developed from, and are more applicable to, out-patient treatment settings.

### Younger cases

Among children and adolescents who have had anorexia nervosa for a relatively short period of time, specific types of family-based treatment (FBT) have a good recovery rate, particularly by the time of follow-up ^7^. However, among younger anorexia nervosa cases, there is some evidence that this superiority over individual therapy is not maintained by the time of follow-up ^8^. Regardless, it remains possible to conclude that FBT is superior to individual approaches in terms of either speed or level of recovery. There are suggestions that this approach can be delivered in fairly diverse ways (e.g., fewer sessions, in multi-family settings) as long as the core therapeutic elements remain in place (e.g., the family taking charge of the patient’s eating).

Other individual-based approaches have been tested with this age group in recent years—particularly, cognitive-behavioural therapy (CBT). There is now evidence that CBT can be a useful approach for adolescents with either underweight or non-underweight eating disorders ^9– 11^. However, it should be noted that FBT has the more immediate benefit when compared directly with CBT for adolescents with bulimia nervosa, although it was not statistically superior to CBT at follow-up ^12^. Therefore, in this age group, CBT should be considered as an alternative that can be used only where FBT is not possible or indicated or where FBT has failed to be effective. There is also a need for further exploration of methods suited to childhood cases.

### Adult cases

***The role of cognitive-behavioural therapy.*** The most powerful additional evidence that has emerged in the past five years is a series of articles that reinforce and extend the place of CBT as the leading approach in the treatment of eating disorders in adults. CBT in different forms was already established as the front-line treatment for bulimia nervosa and binge eating disorder ^13^. Since then, a series of studies ^14– 19^ using Fairburn’s enhanced form of CBT (CBT-E) have demonstrated the following:

- CBT-E is effective for normal-weight bulimia nervosa and atypical eating disorders; approximately half of patients remit and remain well.
- Patients with anorexia do moderately well with CBT-E (approximately 30% entering treatment recover by the end of out-patient therapy, and a somewhat higher rate by the end of in-patient treatment).
- CBT-E is more effective than interpersonal psychotherapy (IPT) and psychodynamic therapy for normal-weight cases. One study ^20^ has suggested that a focal psychodynamic therapy for anorexia nervosa is as effective as CBT-E by the point of follow-up, but so was the “treatment-as-usual” condition, perhaps because the effects of all the therapies were obscured by a relatively high level of in-patient treatment.

***Caveats.*** These conclusions about CBT need to be considered in the light of certain caveats. First, there is no direct comparison of CBT-E with existing versions of CBT, so it is not clear that CBT-E represents an improvement over existing CBT approaches or simply a wider application of core CBT methods across eating disorders.

Second, CBT-E has changed over time; in its early incarnation, it had two forms (“broad” and “focused”). However, the lack of difference in outcomes across these forms was followed by the more recent adoption of a hybrid version, based on the original ‘focused’ form but incorporating the “mood intolerance” module from the “broad” version ^21^. Therefore, understanding the impact of CBT-E requires clarity about which form is under consideration.

Third, other structured therapies that are based on a cognitive model but include other elements (e.g., affective) can be as effective as CBT-E in non-underweight patients ^22^. There remains the possibility that the level of structure in a therapy is key to good outcomes, perhaps as much as the content.

Finally, the nature of the CBT that is being delivered needs to be considered. For example, one study ^23^ concluded that out-patient CBT was not effective for delivering remission in long-standing anorexia nervosa cases (although the chronicity of the individuals’ disorders was not greater than that of some patients in other studies). However, although the chronicity of eating disorders is related to the likelihood of spontaneous recovery ^24, 25^, the impact of chronicity on treatment outcome has not yet been proven ^26^. Possibly more importantly, the comparability of this variant of CBT with others is limited by the fact that the researchers de-emphasised weight gain as a target of treatment, making it secondary and dependent on the patient’s enthusiasm to engage in it. Thus, the conclusion that CBT is not effective in longer-standing cases is not yet proven, as the key outcome variable of weight gain ^27^ was replaced with a primary outcome of improved quality of life.

***Other therapy developments for adults with anorexia nervosa.*** Though better than they were five years ago, CBT’s outcomes for anorexia nervosa remain disappointing. However, that disappointment needs to be understood in the context of the even poorer outcomes of other therapies for anorexia nervosa that have been reported in recent years. These include the following:

- Specialist supportive clinical management (SSCM). Early SSCM findings were promising, suggesting better outcomes than CBT or IPT for anorexia nervosa ^28^. However, those differences disappeared or reversed at long-term follow-up ^29^, suggesting that SSCM might be a therapy that needs to be delivered long-term to maintain its effects. Subsequent studies have suggested a lower recovery rate than for CBT-E ^17^; the out-patient recovery rate was about 15% ^30^.
- The Maudsley model of anorexia nervosa treatment for adults (MANTRA). MANTRA is based on a relatively elaborate theory compared with CBT, on the assumption that CBT is too simplistic to deal with the multiplicity of different pathological factors in anorexia nervosa cases. However, initial findings suggest that it is notably less effective than CBT-E; the recovery rate is similar to that of SSCM ^30^.
- Dialectical behaviour therapy (DBT). A version of this therapy (termed radically open-DBT) has been developed for anorexia nervosa, focusing on the compulsive pathology of such cases. To date, it has been tested only in a clinical case series of in-patients, with a large number of missing data ^31^. According to the method of selecting patients for the final analysis, approximately 15-20% of those entering treatment remitted by the end.

Thus, although the 30% recovery rate for anorexia nervosa when using CBT-E is undoubtedly weaker than for non-underweight cases, it is noticeably stronger than the recovery rates for other therapies, even where pre-treatment characteristics such as age, duration of disorder, and body mass index are comparable. However, most of the therapies outlined above have a reasonable “partial recovery/improvement” rate for anorexia nervosa ^17, 29– 31^, suggesting that each has potential to be developed to be more powerful. Efforts to improve all psychological therapies for anorexia nervosa are as important now as they were a decade ago.

## Adjunctive and symptom-based therapies

It is important to note that there are treatments that are effective at addressing elements of eating pathology, even though they are not expected to produce remission or recovery. Recent key developments in this domain are considered briefly here.

### Nutritional work

Obviously, re-nourishment is not a psychological therapy in itself but is a treatment element that appears to be crucial in facilitating the impact of psychotherapies. Starvation/semi-starvation is a powerful maintaining factor in the eating disorders; it has an impact on biology, cognitions, emotions and social function. Those effects can be seen among normal-weight patients as well as those who are underweight. There is little doubt that restoring nutritional balance is important for recovery, but it has recently been shown that nutritional improvements are important in terms of both positive changes in core cognitions ^32^ and psychosocial functioning, such as quality of life ^33^. Therefore, nutritional changes appear to be necessary for psychotherapies to be effective for eating disorders.

### Cognitive remediation therapy

Cognitive remediation therapy (CRT) is increasingly used to address the cognitive inflexibility that is associated with eating disorders, particularly anorexia nervosa. The evidence to date ^34^ suggests that CRT is associated with greater cognitive flexibility in case series. There is also some evidence from randomised controlled trials that CRT is effective in relieving some aspects of eating pathology and in enhancing retention in other therapies ^35– 37^, although the benefits do not always appear to operate via the expected route of enhanced cognitive flexibility ^34^. The evidence to date is promising, but conclusions will need to await further studies. Two key questions remain to be addressed. First, are the effects of adjunctive CRT associated with positive outcomes from other therapies? Second, is CRT valuable over and above the impact of re-nourishment of the starved patient?

### Support for carers

It is important to remember that those with the eating disorder are not the only people to suffer from its effects. Carers for such patients also experience high levels of stress and distress. Acknowledging carers’ needs has resulted in a range of individual and group support programmes, intended to relieve those experiences. Those interventions are well received ^38^ and are effective in reducing carers’ distress ^39^, although it remains to be determined whether they have any clear benefit in terms of patients’ symptoms.

## Conclusions

There have been substantial developments in the field of psychological therapies for eating disorders since this decade began. To summarise, there has been:

- consolidation of the position of CBT for bulimia nervosa and binge eating disorder ^16, 18, 19^,
- some evidence that other therapies for normal-weight cases can be as effective as CBT ^22^,
- enhanced evidence that FBT is the treatment of choice for younger cases ^7, 8, 12^,
- improvement of the reach of CBT to other eating disorders, including among adolescents ^11, 14– 18^,
- clearer evidence for some adjunctive approaches, even if their target is not recovery ^32– 39^, and
- disappointment that treatment outcomes for adults with anorexia nervosa are still weaker than for non-underweight cases, even though there are differential effects for different therapies ^17, 23, 29– 31^.

Between them, these developments offer both possibilities and challenges. Clinicians have clearer guidance as to what is likely to be effective for their patients, and should be encouraged to work with that information (in the absence of any clear heuristics for treatment matching, apart from age). There remain substantial deficits in our treatment of eating disorders, particularly for cases of anorexia nervosa. Although the development of therapies such as MANTRA and radically open-DBT has been important, at present their benefits are not yet comparable to those of FBT and CBT. CBT and FBT themselves will need further development (e.g., recent evidence that a planful response to a lack of early change is beneficial in FBT for adolescents with anorexia nervosa ^40^). Additionally, other therapies will need to be developed and tested further over the next decade, particularly where they show some promise already in terms of symptom reduction and partial or complete recovery ^22, 28, 30, 31, 34^.
